# Nucleic acid testing identifies high prevalence of blood borne viruses among approved blood donors in Mozambique

**DOI:** 10.1371/journal.pone.0267472

**Published:** 2022-04-28

**Authors:** Nédio Mabunda, Orvalho Augusto, Ana Flora Zicai, Ana Duajá, Sandra Oficiano, Nalia Ismael, Adolfo Vubil, Tufária Mussá, Milton Moraes, Ilesh Jani

**Affiliations:** 1 Instituto Nacional de Saúde, Marracuene, Mozambique; 2 Laboratório de Hanseníase, Instituto Oswaldo Cruz, FIOCRUZ, Brasil; 3 Faculty of Medicine, Universidade Eduardo Mondlane, Maputo, Mozambique; 4 Hospital Central da Beira, Sofala, Mozambique; 5 Hospital Central de Maputo, Maputo, Mozambique; Centers for Disease Control and Prevention, UNITED STATES

## Abstract

**Background:**

Although blood transfusion is an intervention that saves lives, it poses significant risks to the blood receivers, including the transmission of bloodborne pathogens. We aimed at determining the prevalence of Human Immunodeficiency Virus (HIV), Hepatitis B virus (HBV), and Hepatitis C virus (HCV) in candidates approved for blood donation, and in samples considered to be negative in reference blood banks in Mozambique.

**Methods:**

A cross-sectional study was performed between November 2014 and October 2015 in Maputo and Beira cities. Demographic information was obtained from all consenting blood donors using a structured questionnaire. Plasma samples were screened for HIVAb/Ag combinations, HBsAg and Anti-HCV. Blood donors considered to be negative by serological testing were re-tested in pools of six plasma samples using nucleic acid testing (NAT).

**Results:**

Most blood donors were male 2,320 (83.4%) with an age range of 18 to 34 years. The overall seroprevalence of HIV, HBV and HCV infections among blood donors approved for donation was 4.6% (127; 95% CI 3.8–5.4), 4.5% (124; 95% CI 3.7–5.3) and 0.4% (11; 95% CI 0.2–0.7), respectively. The overall frequency by NAT of HIV RNA, HBV DNA, and HCV RNA in serologically negative blood donor samples was 2.6 per 1000 blood donors (7; 95% CI 1.1–5.4); 12.5 per 1000 blood donors (33; 95% CI 8.6–17.5) and 2.6 per 1000 blood donors (6; 95% CI 1.0–5.7), respectively.

**Conclusion:**

Our results show high seroprevalence of HIV and HBV infections in blood donors approved for donation, and high frequency of molecular biomarkers of HIV, HBV, and HCV in blood considered to be safe. These results suggest the need for a new blood screening policy in Mozambique, including the use of NAT to detect infectious blood donations during the immunologically negative window.

## Introduction

Transmission of bloodborne infections such as Human Immunodeficiency Virus (HIV), Hepatitis B virus (HBV), Hepatitis C virus (HCV) and other emerging pathogens to blood receivers remains a current threat [1]. Pre-screening of blood donors, use of blood from regular donors and screening of donated blood for transfusion-transmissible agents prevalent regionally contribute to safer transfusion practices [2].

Most African countries use only serological tests to screen for infectious agents in blood donors. Among serological tests, rapid tests have been the most used due to their low cost and easy management. The use of serological tests increases the transfusion risk due to the long diagnostic window period and lower sensitivity than nucleic acid testing (NAT) [3,4]. These shortcomings are of greater relevance in sub-Saharan Africa due to the relatively high incidence of blood-borne agents [5]. African countries that have introduced NAT for screening for infectious agents have shown a significant reduction in transfusion risk for HIV, HBV and HCV [6,7].

Mozambique has one of the highest HIV prevalence globally, with 13.2% of the population between 15–49 years old infected [8]. In addition, several studies conducted on Mozambican blood donors have reported HBsAg prevalences between 6.0% and 10.6% [9–11]. A higher prevalence of HBsAg (12.2%) has also been reported in younger healthy people aged 18–24 years in southern Mozambique [12]. HCV infection has been reported with prevalences of 1.2% and 1.0% in men and women, respectively, in a Blood Bank in the capital city Maputo [10].

Blood banks in Mozambique have considerably improved the pre-screening of blood donors and testing for blood-borne pathogens. Serological screening of HIV, HBV, HCV and *Treponema pallidum* is mandatory in blood banks before blood donation, but NAT is not performed routinely. However, the risk of transmission of bloodborne pathogens is unknown.

This study aimed to determine the seroprevalence of HIV, HBV and HCV infections in two reference blood banks in the south and central regions of Mozambique. The results show high seroprevalence of HIV and HBV in candidates approved for blood donation, and high frequency of nucleic acid of these viruses in samples considered negative by the current testing protocols.

## Materials and methods

### Study design and population

A cross-sectional study was performed between November 2014 and October 2015 at the Blood Bank of *Hospital Central de Maputo* (South of Mozambique) and *Hospital Central da Beira* (Center of Mozambique).

Participants of this study were recruited according to the inclusion criteria established by the National Blood Service. All potential donors must respond to a standardised survey with 22 questions (six leading to definitive exclusion and 16 to temporary exclusion for blood donation). Being older than 65 years of age, having a chronic disease, or being at risk of having a sexually transmitted infection constitute criteria for definitive exclusion. Temporary exclusion criteria include lack of knowledge about infection transmission by blood, having other illnesses, or having an abnormal physiological condition not compatible with a donation (medication, pregnancy, recent vaccination, or others).

In addition to the above risk behavior survey, the general health status of blood donor candidates is assessed by parameters such as weight, hemoglobin, blood pressure, and signs of an infectious disease.

**Fig 1:**
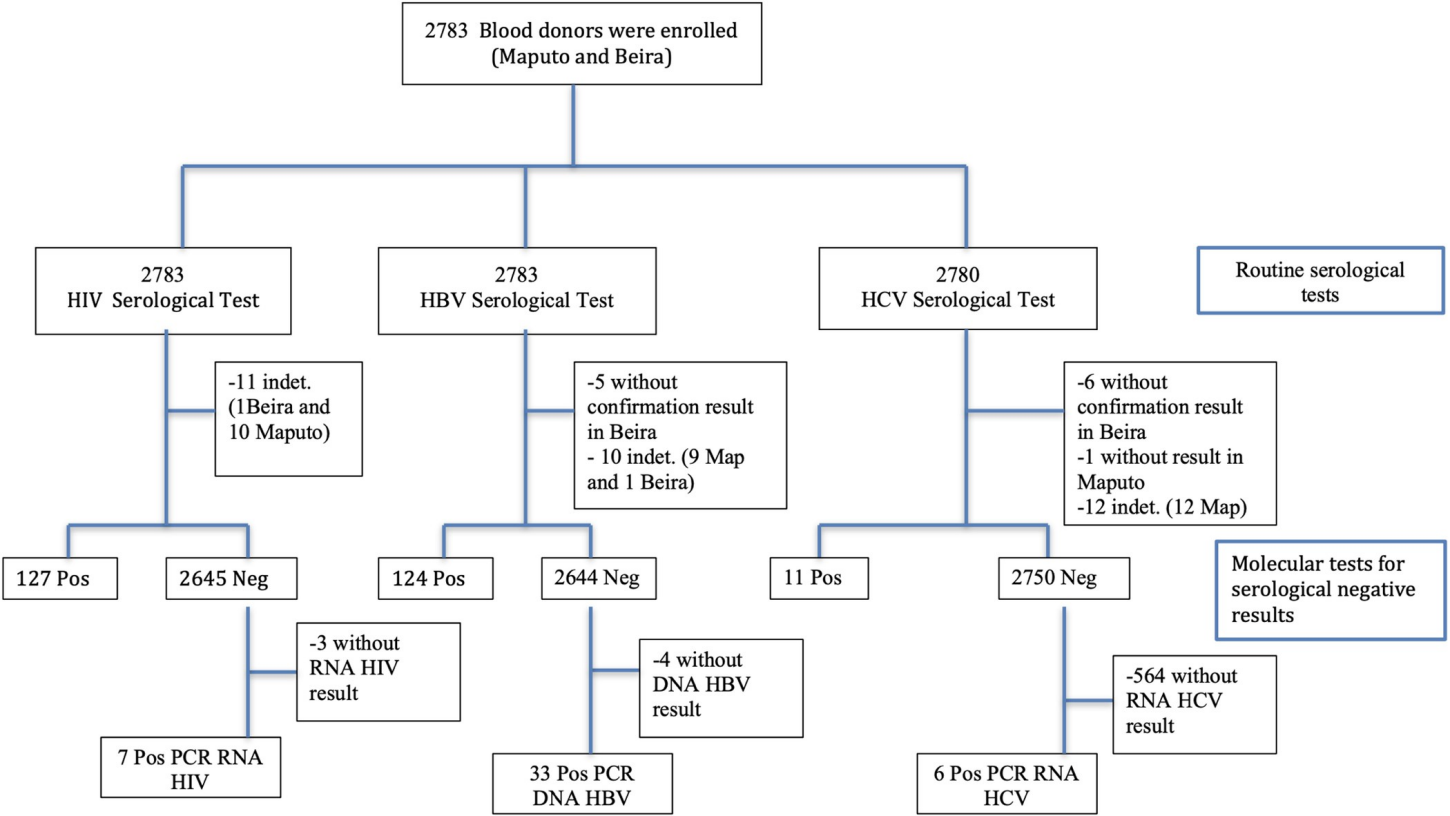
Flow diagram of study participants. All 2,783 study participants were tested for HIV, HBV, and HCV serology. Samples with negative results in serology were submitted to molecular testing for the corresponding virus.

After inclusion, a blood bag unit was collected from each participant for the blood bank routine (testing and transfusion). In addition, 9 mL of whole blood was collected into a Vacuum tube with K3EDTA for study purposes. From the latter, plasma was separated and stored at -20°C for serological and molecular tests. All samples were screened for HIV Ab/Ag combinations, HBsAg and anti-HCV at the blood bank, following sequential algorithms of two commercial kits as described below. Molecular tests for HIV, HBV and HCV were carried out at the Instituto Nacional de Saúde (Fig 1).

### Clinical and demographic data

Demographic information of study participants (age, sex, nationality, type of donor) was obtained from all consenting blood donors using a structured questionnaire (S1 File).

### Serological tests

The screening of blood samples for HIV was done using a qualitative enzyme immunoassay GENSCREEN PLUS HIV Ag-Ab (Bio-Rad, Marnes-l Coquette, France), a test with 100% sensitivity and 99.95% specificity to detect both HIV antibody and HIV-1 p24 antigen. As per the national HIV testing algorithm, reactive samples were re-tested using the UNIGOLD (Trinity Biotech Plc, Bray, Co. Wicklow, Ireland) rapid test with 100% sensitivity and 100% specificity to detect antibodies to HIV-1 and/or HIV-2. HBsAg screening was performed using the Advanced Quality HBsAg ELISA Test Kit (InTec Products, INC, China) with 99.9% sensitivity and 100% specificity. Reactive samples were confirmed using the Advanced Quality HBsAg Rapid Test (InTec Products, INC, China) with 100% sensitivity and 100% specificity. Finally, the screening of HCV was done using the Advanced Test Kit HCV (InTec Products, INC, China) with 99.0% sensitivity and 99.8% specificity for anti-HCV, and confirmed with Advanced Quality Rapid Anti-HCV (InTec Products, INC, China) with 96.3% sensitivity and 100% specificity for anti-HCV detection.

Samples were considered negative if non-reactive in the first test. Samples were considered positive if reactive in the first and second tests. Finally, samples were considered indeterminate if the results were discordant.

### DNA and RNA Quantification and Detection

Samples with negative serology for HIV, HBV and HCV were submitted to nucleic acid testing for the same agents. This molecular testing was performed in pools of six plasma samples using the COBAS AmpliPrep/COBAS TaqMan HIV Test, v2.0 for HIV, COBAS AmpliPrep/COBAS TaqMan HBV Test, v2.0 for HBV, and Roche Cobas AmpliPrep/Cobas TaqMan HCV Quantitative test, version 2.0 for HCV (all from Roche Molecular Systems, Inc., Branchburg, NJ, USA) with a detection limit of 20 IU/mL. If nucleic acid was detected in any tested pools, then all pool samples were retested individually using the same kits.

### Statistical analysis

Data from all study participants were collected in a Microsoft Excel file and then exported to Stata 15 (StataCorp. 2017. Stata: Release 15. Statistical Software. College Station, TX: StataCorp LLC) for analysis. For categorical variables, descriptive analysis was performed and the data was summarized in proportions and frequency tables. Means with their respective standard deviations and ranges were used to summarize continuous variables. For proportions of positivity exact 95% confidence interval was reported. The frequencies of serological markers are presented as percentages, whereas for RNA markers these are presented per 1000. The missing and indeterminate results were excluded from the analysis of each marker. The analysis was stratified by site.

A two-step procedure was used to compute the potential number of infected blood donations in each blood bank. First, the proportion of positive molecular tests among tested (P_RNA+_) was estimated as the product of the proportion of negatives to serology (P_ser-_) and the proportion of positive molecular test among the negative to serology (P_RNA+ser-_):

$$P_{RNA+}=P_{ser-}∙P_{RNA+ser-}$$

The details to compute the confidence interval for the P_RNA+_ are explained in the supplementary materials (S2 File).

The second step is the multiplication of P_RNA+_ and its confidence interval limits to the number of blood donors in each year between 2015 and 2020. This provides the estimated numbers of serologically negative blood donors infected with HIV, HBV or HCV.

#### Ethical considerations

The study protocol was approved by the national health bioethics committee in Mozambique (263/CNBS/2014). All procedures were carried out following the Helsinki Declaration as revised in 2013. Written informed consent was obtained from all blood donors before enrolment in the study.

## Results

### Serological analysis for anti-HIV antibodies, HBsAg, and anti-HCV antibodies

A total of 2,783 blood donors participated in the study, 1,502 from Maputo and 1,281 from Beira (Fig 1). Most blood donors were young males aged 18 to 34 years, had a secondary education level and were single (Table 1). Replacement donors accounted to around three quarters of donors at the Maputo Central Hospital (1,121; 74.6%), while at the Beira Central Hospital about a third were of this donor type (487; 38.0%).

**Table 1:** Sociodemographic characteristics of blood donors.

Characteristics	Maputo Central Hospital N (%)	Beira Central Hospital (N (%)
**Total of participants**	1502 (100)	1281 (100)
**Sex**		
Male	1174(78.2)	1146 (89.5)
Female	328 (21.8)	132 (10.3)
Missing	0(0.0)	03 (0.2)
**Age (years)**		
<18	9 (0.6)	53 (4.1)
18–24	372 (24.8)	492 (38.4)
25–34	564 (37.5)	421 (32.9)
35–44	323 (21.5)	170 (13.3)
45–65	233 (15.5)	121 (9.4)
Missing	1 (0.1)	24 (1.9)
Min-Max	16–65	16–65
Mean (SD)	32.8(10.57)	28.9 (10.00)
**Education**		
None	16 (1.1)	6 (0.5)
Primary	330 (22.0)	115 (9.0)
Secondary	990 (65.9)	1042 (81.3)
Higher	166 (11.0)	106 (8.3)
Missing	0 (0.0)	12 (0.9)
**Donation type**		
Regular	381 (25.4)	765 (59.7)
Replacement	1121 (74.6)	487 (38.0)
Missing	0 (0.0)	29 (2.3)
**Marital Status**		
Single	1159 (77.2)	1025 (80.0)
Married	322 (21.4)	239 (18.7)
Divorced/Widow	15 (1.0)	5 (0.4)
Missing	6 (0.4)	12 (0.9)

The overall seroprevalence of HIV infection among study participants was 4.6% (127 infections; 95% Confidence Interval: 3.8–5.4; Table 2), being higher in Maputo with 5.6% (83; 95% CI: 4.5–6.8) than in Beira with 3.4% (44; 95% CI 2.5–4.6). The seroprevalence of HBsAg was 4.5% (124; 95% CI 3.7–5.3), similar in both blood banks with 4.5% (67; 95% CI: 3.5–5.7) in Maputo and 4.5% (57; 95% CI:3.4–5.8) in Beira. The seroprevalence of HCV antibodies was 0.4% (11; 95% CI 0.2–0.7). This was higher in Maputo with 0.5% (7; 95% CI: 0.2–1.0) than in Beira with 0.3% (4; 95% CI 0.1–0.8).

**Table 2:** Frequency of infectious serological markers in blood donors.

Characteristics	Maputo Central Hospital			Beira Central Hospital			Total		
	Tested*	Positive	%(95CI)	Tested*	Positive	%(95CI)	Tested*	Positive	%(95CI)
**All participants**									
Anti-HIV	1492	83	5.6 (4.5–6.8)	1280	44	3.4 (2.5–4.6)	2772	127	4.6 (3.8–5.4)
HBsAg	1493	67	4.5 (3.5–5.7)	1275	57	4.5 (3.4–5.8)	2768	124	4.5 (3.7–5.3)
Anti-HCV	1489	7	0.5 (0.2–1.0)	1275	4	0.3 (0.1–0.8)	2764	11	0.4 (0.2–0.7)
HBsAg or Anti-HIV†	1483	145	9.8 (8.3–11.4)	1274	96	7.5 (6.1–9.1)	2757	241	8.7 (7.7–9.9)
Anti-HCV or Anti-HIV†	1479	85	5.7 (4.6–7.1)	1274	45	3.5 (2.6–4.7)	2753	130	4.7 (4.0–5.6)
HBsAg or Anti-HCV†	1480	74	5.0 (3.9–6.2)	1270	61	4.8 (3.7–6.1)	2750	135	4.9 (4.1–5.8)
Anti-HCV or Anti-HIV or HBsAg †	1470	147	10.0 (8.5–11.6)	1269	98	7.7 (6.3–9.3)	2739	245	8.9 (7.9–10.1)
HBsAg and Anti-HIV†	1483	5	0.3 (0.1–0.8)	1274	4	0.3 (0.1–0.8)	2757	9	0.3 (0.1–0.6)
HBsAg and Anti-HCV†	1480	0	< 0.2	1270	0	< 0.3	2750	0	< 0.1
Anti-HCV and Anti-HIV†	1479	2	0.1 (0.0–0.5)	1274	0	< 0.3	2753	2	0.1 (0.0–0.3)
**Regular Donors**									
Anti-HIV	377	10	2.7 (1.3–4.8)	764	35	4.6 (3.2–6.3)	1141	45	3.9 (2.9–5.2)
HBsAg	379	7	1.8 (0.7–3.8)	760	28	3.7 (2.5–5.3)	1139	35	3.1 (2.1–4.2)
Anti-HCV	374	2	0.5 (0.1–1.9)	760	1	0.1 (0.0–0.7)	1134	3	0.3 (0.1–0.8)
HBsAg or Anti-HIV†	375	16	4.3 (2.5–6.8)	759	59	7.8 (6.0–9.9)	1134	75	6.6 (5.2–8.2)
Anti-HCV or Anti-HIV†	370	10	2.7 (1.3–4.9)	759	33	4.3 (3.0–6.1)	1129	43	3.8 (2.8–5.1)
**Replacement Donors**									
Anti-HIV	1115	73	6.5 (5.2–8.2)	487	7	1.4 (0.6–2.9)	1602	80	5.0 (4.0–6.2)
HBsAg	1114	60	5.4 (4.1–6.9)	486	29	6.0 (4.0–8.5)	1600	89	5.6 (4.5–6.8)
Anti-HCV	1115	5	0.4 (0.1–1.0)	486	3	0.6 (0.1–1.8)	1601	8	0.5 (0.2–1.0)
HBsAg or Anti-HIV†	1108	129	11.6 (9.8–13.7)	486	35	7.2 (5.1–9.9)	1594	164	10.3 (8.8–11.9)
Anti-HCV or Anti-HIV†	1109	75	6.8 (5.4–8.4)	486	10	2.1 (1.0–3.8)	1595	85	5.3 (4.3–6.5)

*Indeterminates are excluded from the analysis.

†The combinations exclude cases with one antibody information.

95%CI -Binomial Exact Confidence Interval.

The detection of either anti-HCV or anti-HIV antibodies was reported in 4.7% of blood donors (130; 95% CI 4.0–5.6), being highest in Maputo with 5.7% (85; 95% CI 4.6–7.1) than in Beira with 3.5% (45; 95% CI 2.6–4.7). The detection of either HBsAg or anti-HIV antibodies was reported in 8.7% of blood donors (241; 95% CI 7.7–9.9), being higher in Maputo with 9.8% (145; 95% CI 8.3–11.4) than in Beira with 7.5% (96; 95% CI 6.1–9.1).

The presence of either anti-HIV antibodies, HBsAg, or Anti-HCV antibodies was detected in 8.9% of study participants (245; 95% CI 7.9–10.1). This was higher in Maputo at 10.0% (147; 95%CI 8.5–11.6) than in Beira at 7.7% (98; 95% CI 6.3–9.3).

The simultaneous detection of HBsAg and anti-HIV antibodies was reported in 0.3% of blood donors (9; 95% CI 0.1–0.6), whereas anti-HCV and anti-HIV antibodies were simultaneously present in the plasma of 0.1% of study participants (2; 95% CI 0.0–0.3).

### Molecular analysis of HIV, HBV and HCV in seronegative samples

The overall frequency of HIV RNA in antibody-negative blood donor samples was 2.6 per 1000 blood donors (7 infections; 95% CI 1.1–5.4; Table 3), being higher in Beira at 4.9 per 1000 blood donors (6; 95%; CI 1.8–10.5) than in Maputo at 0.7 per 1000 blood donors (1; 95% CI 0.0–3.9). The overall frequency of HBV DNA was 12.5 per 1000 blood donors (33 infections; 95% CI 8.6–17.5), with higher frequency observed in Beira at 13.2 per 1000 blood donors (16; 95% CI 7.6–21.3) than in Maputo at 11.9 per 1000 blood donors (17;95% CI 7.0–19.1). The frequency of HCV RNA was 2.6 per 1000 blood donors (6 infections; 95% CI 1.0–5.7), being higher in Beira 5.0 per 1000 blood donors (4; 95% CI 1.4–12.6) than in Maputo 1.4 per 1000 blood donors (2; 95% CI 0.2–4.9).

**Table 3:** Frequency of HIV RNA, HBV DNA and HCV RNA markers in a serologically negative blood sample.

Characteristics	Maputo Central Hospital			Beira Central Hospital			Total		
	Tested*	Positive	Per 1000 (95CI)	Tested*	Positive	Per 1000 (95CI)	Tested*	Positive	Per 1000 (95CI)
NAT RNA HIV	1409	1	0.7 (0.0–3.9)	1236	6	4.9 (1.8–10.5)	2645	7	2.6 (1.1–5.4)
NAT DNA HBV	1423	17	11.9 (7.0–19.1)	1213	16	13.2 (7.6–21.3)	2636	33	12.5 (8.6–17.5)
NAT RNA HIV or NAT DNA HIV	1336	18	13.5 (8.0–21.2)	1173	19	16.2 (9.8–25.2)	2509	37	14.7 (10.4–20.3)
NAT RNA HCV	1478	2	1.4 (0.2–4.9)	808	4	5.0 (1.4–12.6)	2286	6	2.6 (1.0–5.7)
NAT any on negative to all serology†	1323	20	15.1 (9.3–23.3)	1171	23	19.6 (12.5–29.3)	2494	43	17.2 (12.5–23.2)

* Only negatives to serology are included.

† All negatives are kept regardless of some NAT missing (are considered negative).

The frequency of either HIV RNA or HBV DNA was 14.7 per 1000 blood donors (37; 95% CI 10.4–20.3), with higher frequency observed in Beira 16.2 per 1000 blood donors (19; 95% CI 9.8–25.2) than in Maputo 13.5 per 1000 blood donors (18; 95% CI 8.0–21.2). The frequency of nucleic acid of either of three infectious agents in serologically negative samples was 17.2 per 1000 blood donors (43 infections; 95% CI 12.5–23.2), with higher frequency observed in Beira 19.6 per 1000 blood donors (23; 95% CI 12.5–29.3) than in Maputo 15.1 per 1000 blood donors (20; 95% CI 9.3–23.3).

The median viral load in HIV positive RNA samples was 89 000 copies/ml (7 711–4 744 500). The median viral load for HBV positive DNA samples was 31 IU/mL (<20–986 035) and the median viral load for HCV Positive RNA samples was 14 810 IU/mL (<20–5 960 000).

### Estimated numbers of serologically negative blood donors infected with HIV, HBV or HCV in 2015 to 2020

For each of the two blood banks, we estimated the number of donors infected with either of the three viruses that would have been missed by serological testing (Table 4). In the years 2015 to 2020 at the Maputo Central Hospital, we estimate that an average of 347.5 (328–382) individuals positive for one of the three viruses was approved to donate blood each year. In the same period at the Beira Central Hospital, we estimate that an average of 114.2 (75–134) donors positive for one of the three viruses was yearly approved to donate blood.

**Table 4:** Estimated numbers of serologically negative blood donors infected with HIV, HBV or HCV in 2015–2020.

Year	Blood Donors	PE	LB	UB
**Maputo**				
2015	28,112	382	216	548
2016	24,558	334	189	479
2017	24,301	330	187	474
2018	26,230	357	202	511
2019	26,049	354	201	508
2020	24,082	328	185	470
Total	153,332	2085	1714	2455
**Beira**				
2015	7,141	129	77	181
2016	7,358	133	79	187
2017	7,424	134	80	189
2018	6,402	116	69	163
2019	4,123	75	45	105
2020	5,403	98	58	137
Total	37,851	685	570	800

PE-point estimate.

LB-Lower bound of 95% confidence interval.

UB-Upper bound of 95% confidence interval.

## Discussion

Screening blood donors using increasingly sensitive laboratory assays, with a shorter diagnostic window period, has decreased the global risk of transmission of infectious agents during blood donation [2]. Nevertheless, the progress in sub-Saharan Africa has been slower [13]. In Mozambique, the background risk of blood-borne viral infections is substantial due to their high prevalence in the donor population [9–11]. In this study, we document the prevalence of HIV, HBV, and HCV in individuals approved for blood donation and the risk of blood contamination by these three agents.

The prevalence of HIV infection reported in this study was similar to that found in blood banks from Mozambique from 2014 to 2016 [13]. The prevalence of HIV infection among potential blood donors in Maputo was higher than in Beira. This observed difference is probably due to the higher prevalence of HIV in the general population in Maputo City (16.9%) and Province (22.9%) when compared to Beira (16.3%) [3], and is compounded by the pre-screening strategy used in Beira.

Several studies reported that regular blood donors have a lower prevalence of HIV, HBV and HCV infections when compared to replacement blood donors [9–11,14,15]. Accordingly, our data showed that regular donors in Maputo had a lower prevalence of HIV and HBV when compared to replacement donors. On the other hand, regular donors in Beira had a higher prevalence of the three viruses. This may be due to the use of rapid testing for HIV pre-screening among replacement blood donors in Beira, instead of the routine risk survey administration used in Maputo. Blood donor pre-screening with rapid diagnostic tests, involving regular and replacement donors, has outlined an efficient strategy in reducing the risk of bloodborne transmission of infectious agents and reducing overall testing costs [16]. This strategy, in combination with NAT and pathogen reduction or pathogen inactivation, has shown a substantial impact in reducing the risk of bloodborne transmission of infectious agents [17,18]. Another factor that may explain the higher prevalence of HIV and HBV in regular blood donors in Beira could be the inclusion of many first-time donors, as these carry the same risk of harbouring blood-borne infectious agents when compared to replacement donors [19].

Although the prevalence of the three viruses in our study was lower than in the general population, these were still far higher than the 1% or less recommended by WHO for approved blood donors [2]. The selection of safe donors and their retention is an important measure for transfusion safety [2,20,21]. To effectively reduce the transmission of infectious agents from a blood transfusion, the pre-screening process needs to be adapted to the epidemiological, cultural, and appropriate type of population [22,23]. Stokx et al. 2011, pointed out that the deficiency of the pre-screening process compromises the exclusion of higher risk blood donors for the transmission of infectious agents in Mozambique [9]. Our results reinforce the need to improve the pre-screening to effectively exclude as many high-risk blood donors as possible.

Using molecular testing, we reported a high frequency of HIV, HBV and HCV in blood donors approved for blood donation by the routine screening process in two blood banks. For HIV and HCV, this escape from the serological screening process may be linked to the diagnostic window period as explored in several studies [24,25]. For HBV, beyond the window period, the non-detection of surface antigen (HBsAg) may be linked to occult Hepatitis B infections [26–28]. Our NAT results in HBsAg negative samples showed occult Hepatitis B virus frequency of 0.98% (14/16) and a frequency of samples in immunological window period of 0.13% (2/16). These results reveal the relevance of occult hepatitis B as a contemporary risk during blood transfusion in Mozambique [29].

Despite the high frequency of HIV, HBV and HCV detected by NAT in our study, the mini-pool strategy may have underestimated the real frequency of viruses in negative samples. Studies show that the window period using NAT in individual testing was reduced by 31% for HIV and 91% for HCV and HBV, while when using mini-pools of 16 samples the reduction was 17% for HIV and 87% for HCV and HBV [30,31]. It would, however, be expected that the use of mini-pools of six samples, as employed in this study, would substantially reduce the risk of transmission during the window period.

Our results highlight the ongoing contribution of blood transfusion to the incidence of HIV, HBV and HCV in Mozambique. However, the findings of this study should be viewed with caution because: 1) We observed the risk of infection at the level of blood donors but a blood bag has several components (platelets, whole blood, plasma, and others), which can be administrated to different people thus multiplying the risk of infection; 2) The data cannot be generalized as the study was carried out in two major national reference blood banks, where quality standards are in place and ELISA tests are used to screen blood donors. Most other testing sites for blood donors in Mozambique use rapid tests with a longer diagnostic window period, and potentially increased risk of transmission of bllod-borne infections; 3) The initial serological testing was done using the blood bank algorithm, which may have influenced the seroprevalence of the three agents.

## Conclusion

Our results show a high seroprevalence of HIV and HBV infections in blood donors approved for donation, and high frequency of molecular biomarkers of HIV, HBV and HCV in blood considered to be safe in two reference Blood Banks of Mozambique. These results highlight the need for a new blood screening policy in Mozambique, including the use of NAT to detect infectious blood donations during the immunologically negative window.

## Supporting information

S1 File Questionnaire for participants. (DOCX)

S2 File Estimating the number of infected blood donations in each blood bank. (DOCX)
