# Supplementary material for: Nucleic acid testing identifies high prevalence of blood borne viruses among approved blood donors in Mozambique
Source: PLoS One. 2022 Apr 28;17(4):e0267472. doi: 10.1371/journal.pone.0267472 (PMC9049559; doi:10.1371/journal.pone.0267472)
Supplement: S1 File — (DOCX) [file pone.0267472.s001.docx]

**MINISTÉRIO DA SAÚDE**

**Instituto Nacional de Saúde de Moçambique**

# Departamento de Imunologia

**“Risco de Transmissão de HIV, HBV, HCV por Transfusão Sanguínea em Moçambique”**

Dados sócio-demográficos

| Número do Dador: \|___\|___\|___\|___\|___\|___\|___\|___\|___\|  Número de Frasco  \|___\|___\|___\|___\|___\|___\|___\|___\|___\| | Idade: \|___\|___\| anos  Tipo de Dador:  1.Repositor  2. Voluntário |
| --- | --- |
| Sexo:  1. Masculino:  2. Feminino: | Grau de Escolaridade:  1. Analfabeto:  2. Primário:  3. Secundário / Técnico :  4. Superior: |
| Estado civil:  1. Solteiro/a:  2. Casado/a:  3. Divorciado/a:  4. Viúvo/a: | Cor da pele:  1. Negra:  2. Mista:  3. Branca: |
| Local de nascimento:  Local de Residência (província): | Profissão: |

Observações do entrevistador: _______________________________________________________________________________________________________________________________________________________________________________________________________________________________________________________________

Maputo, _________ (Dia) de ___________________(Mês) de_______(ano)

_________________________________________________

(Assinatura do Dador)

_________________________________________________

(Assinatura do entrevistador)

**MINISTRY OF HEALTH**

**Instituto Nacional de Saúde of Mozambique**

# Departamento de Imunologia

**“Risk of HIV, HBV and HCV transmission by blood transfusion in Mozambique”**

Socio-Demographic data

| Donor number: \|___\|___\|___\|___\|___\|___\|___\|___\|___\|  Tube number  \|___\|___\|___\|___\|___\|___\|___\|___\|___\| | Age: \|___\|___\| years  Donation type:  1.Replacement  2. Regular |
| --- | --- |
| Sex:  1. Male:  2. Female: | Education:  1. None:  2. Primary:  3. Secondary:  4. Higher: |
| Marital Status:  1. Single:  2. Married:  3. Divorced:  4. Widow: | Skin color:  1. Black:  2. Mixed:  3. White: |
| Place of birth:  Place of residence (provínce): | Occupation: |

Interviewer’s observation: ______________________________________________________________________________________________________________________________________________________________________________________________________________________________________________________________

Maputo/Beira, _________ (Day) of ___________________ (Month) _________year

_________________________________________________

(Donor Signature)

_________________________________________________

(Interviewer’s Signature)
