# Supplementary material for: Nucleic acid testing identifies high prevalence of blood borne viruses among approved blood donors in Mozambique
Source: PLoS One. 2022 Apr 28;17(4):e0267472. doi: 10.1371/journal.pone.0267472 (PMC9049559; doi:10.1371/journal.pone.0267472)
Supplement: S2 File — (DOCX) [file pone.0267472.s002.docx]

**Appendix**

**Estimating the number of infected blood donations in each blood bank**

A two-step procedure was used to compute the potential number of infected blood donations in each blood bank.

First, the proportion of positive molecular test among tested (P_RNA+_) was estimated as the product of the proportion of negatives to serology (P_ser-_) and the proportion of positive molecular test among the negative to serology (P_RNA+ser-_):

[1]

$$P_{RNA+}=P_{ser-}\cdot P_{RNA+ser-}$$

The second step was to multiply this proportion to the number of blood donors in each year.

In order to compute the confidence intervals for P_RNA+_ we used two approaches. The first, analytic, we converted equation 1 into

$$\log P_{RNA+}=\log P_{ser-}+ \log P_{RNA+ser-}$$

From this, the variance is obtained as^[[1]](#footnote-1)^

$$var(\log P_{RNA+})=var(\log P_{ser-})+ var(\log P_{RNA+ser-})$$

Applying the delta method to compute the variance to each of the proportions on the right we obtain

$$var(\log P_{RNA+})=\left( \frac{S_{ser-}}{P_{ser-}} \right)^{2}+ \left( \frac{S_{RNA+ser-}}{P_{RNA+ser-}} \right)^{2}$$

where the S are standard errors.

The second approach to estimate the confidence interval for P_RNA+_ is to use a Monte Carlo simulations. We simulated 1000 P_ser-_ and P_RNA+ser-_ assuming that each proportion raise from normal distributions. Therefore, we obtained 1000 realizations of the P_RNA+_ from which we take the 2.5 and 97.5^th^ percentiles as the confidence intervals limits.

Table 1- the proportion of positive molecular test among tested (P_RNA+_) using the analytic and Monte Carlo approach for the confidence intervals

|  | **Negative serology test** | **Positive molecular test among negative** | **Positive molecular test among tested**  **[Analytic]** | **Positive molecular test among tested**  **[Monte Carlo]** |
| --- | --- | --- | --- | --- |
|  | **% (95CI)** | **per 1000 (95CI)** | **per 1000 (95CI)** | **per 1000 (95CI)** |
| Maputo City | 90.0 (88.4 - 91.5) | 15.1 (9.3 - 23.3) | 13.6 (8.6 - 20.6) | 13.6 (7.7 - 19.5) |
| Beira City | 92.3 (90.7 - 93.7) | 19.6 (12.5 - 29.3) | 18.1 (11.8 - 26.6) | 18.1 (10.8 - 25.4) |

1. Please note that P_ser-_ is independent of P_RNA+ser-_ [↑](#footnote-ref-1)
